# Complicate dynamical properties of a discrete slow-fast predator-prey model with ratio-dependent functional response

**DOI:** 10.1038/s41598-023-45861-2

**Published:** 2023-11-23

**Authors:** Xianyi Li, Jiange Dong

**Affiliations:** Department of Big Data Science, School of Science, Zhejiang University of Science and Technology, Hangzhou, 310023 China

**Keywords:** Ecology, Mathematics and computing

## Abstract

Using a semidiscretization method, we derive in this paper a discrete slow-fast predator-prey system with ratio-dependent functional response. First of all, a detailed study for the local stability of fixed points of the system is obtained by invoking an important lemma. In addition, by utilizing the center manifold theorem and the bifurcation theory some sufficient conditions are obtained for the transcritical bifurcation and Neimark-Sacker bifurcation of this system to occur. Finally, with the use of Matlab software, numerical simulations are carried out to illustrate the corresponding theoretical results and reveal some new dynamics of the system. Our results clearly demonstrate that the system is very sensitive to its fast time scale parameter variable.

## Introduction

In recent decades, human beings are suffering from some disasters of destroying the natural environment, such as pollution, species extinction, virus epidemics, etc. It is important to provide strategies to relieve the environmental pressure. Mathematical modeling can reveal the changing trend of the natural environment, therefore, more and more biologists, ecologists and mathematicians are committed to studying ecological balance using mathematical models.

The classical prey-predator model with prey-dependent functional response as proposed by Holling in 1965^1^ is given by a system of coupled ordinary differential equations:

1 $$\begin{aligned} \left\{ \begin{array}{ll} \frac{dX}{dT}=R(X)X-P(X)Y,\\ \frac{dY}{dT}=eP(X)Y-M(Y)Y, \end{array}\right. \end{aligned}$$

where *X* and *Y* respectively denote the prey and predator densities at time *T*. Here both species are assumed to be distributed homogeneously within their habitats. The function *R*(*X*) represents prey’s per capita growth rate. In this model, the prey-predator interaction is described by the prey-dependent function *P*(*X*) known as functional response, which quantifies the average amount of prey consumed by a single predator per unit of time. The predator is assumed to be specialist. The parameter $e(0< e < 1)$ known as the conversion efficiency determines the fraction of prey biomass that contributes to the predator’s growth. The function *M*(*Y*) represents per capita death rate of predators in the absence of prey. The important assumptions of the classical model are that predator encounters prey at random and the trophic function depends on prey abundance only. However, in the late 1989, Adriti and Ginzburg^2^ challenged the classical theory by showing the importance of predator interference whenever the prey abundance is low. The authors argued that “the trophic function must be considered on the slow time scale of population dynamics at which the models operate-not on the fast behavioral time scale”^2^. It is therefore reasonable to assume that the functional response depends on the ratio of prey to predator abundance rather than just on the prey abundance when the available prey density is low. That is, in order to reflect the predator interference, the per capita functional response should be a function of *X*/*Y* rather than *X*. Based upon this idea, the Michaelis-Menten-Holling type functional response was introduced, also known as ratio-dependent functional response^2^. In this paper we consider the ratio-dependent prey-predator system with the logistic growth in prey as a baseline model as follows

2 $$\begin{aligned} \left\{ \begin{array}{ll} \frac{dX}{dT}=rX \left( 1-\frac{X}{k}\right) -\frac{a XY}{X+gY},\\ \frac{dY}{dT}=\frac{bXY}{X+gY}-mY, \end{array}\right. \end{aligned}$$

where *r* is the linear growth rate of the prey, *k* is the environment carrying capacity to prey, *b* is the maximum per capita growth rate of the predator (note that the corresponding term approaches its limiting value *bY* when *X* becomes very large), *m* is the predator mortality and *g* is the relative saturation factor between the two species.

By rescaling the variables

$$\begin{aligned} x=\frac{X}{k},\quad y=\frac{g}{k}Y,\quad \tau =bT, \end{aligned}$$

we obtain the following dimensionless system

3 $$\begin{aligned} \left\{ \begin{array}{ll} \epsilon \frac{dx}{d\tau }=x-x^2-\frac{\alpha xy}{x+y},\\ \frac{dy}{d\tau }=\frac{xy}{x+y}-\delta y, \end{array}\right. \end{aligned}$$

where the new parameters $\epsilon =\frac{b}{r}$, $\alpha =\frac{a}{gr}$ and $\delta =\frac{m}{b}$ are dimensionless.

Generally, one assumes that the prey population grows faster than the predator population (which, in fact, is often the case in nature). So, we have $b<r$, implying $0<\epsilon <1$.

Note that the dimensionless time $\tau$ in the system (3) is the slow time. With the transformation $t=\frac{\tau }{\epsilon }$, the equivalent system in fast time scale is

4 $$\begin{aligned} \left\{ \begin{array}{ll} \frac{dx}{dt}=x-x^2-\frac{axy}{x+y},\\ \frac{dy}{dt}=\epsilon \left( \frac{xy}{x+y}-\delta y \right) . \end{array}\right. \end{aligned}$$

It is not easy to solve a complicate differential equation (system) without computer. So, one tries to use discretization method to derive and study the discrete model of a complicate differential equation (system) so that one can better understand the properties of corresponding continuous system. How to discretize a complicate differential equation (system)? Many discretization methods, such as the forward Euler method, the backward Euler method, the semidiscretization method, and so on, can be utilized. The discrete version of the system (4) has not been investigated yet. We here use the semidiscretization method to study its discrete model. The advantage for this kind of discrete method is for one not to require to consider the step size. Relatively speaking, this kind of method can reduce the number of parameters so that the system studied is easily investigated.

For this, suppose [*t*] to denote the greatest integer not exceeding *t*. Consider the average change rate of the system (4) at integer number points

5 $$\begin{aligned} \left\{ \begin{array}{ll} \frac{1}{x(t)}\frac{dx(t)}{dt}=1-x([t])-\frac{ay([t])}{x([t])+y([t])},\\ \frac{1}{y(t)}\frac{dy(t)}{dt}=\epsilon \left( \frac{x([t])}{x([t])+y([t])}-\delta \right) . \end{array}\right. \end{aligned}$$

It is easy to see that the system (5) has piecewise constant arguments, and that the solution (*x*(*t*), *y*(*t*)) of the system (5) for $t \in [0, +\infty )$ possesses the following characteristics:

1. on the interval $[0, +\infty )$, *x*(*t*) and *y*(*t*) are continuous;
2. when $t\in [0, +\infty )$ except for the points $t\in \{0, 1, 2, 3, \ldots \}$, $\frac{d x(t)}{dt}$ and $\frac{d y(t)}{dt}$ exist everywhere.

Integrating (5) over the interval [n,t] for any $t\in [n, n+1)$ and $n=0,1,2,\ldots$ obtains the following system

6 $$\begin{aligned} \left\{ \begin{array}{ll} x(t)=x_ne^{(1-x_n)-\frac{ay_n}{x_n+y_n}}(t-n), \\ y(t)=y_ne^{\epsilon \left( \frac{x_n}{x_n+y_n}-\delta \right) }(t-n), \end{array}\right. \end{aligned}$$

where $$x_n=x(n)$$ and $$y_n=y(n)$$.

Letting $t\rightarrow (n+1)^-$ in (6) produces

7 $$\begin{aligned} \left\{ \begin{array}{ll} x_{n+1}=x_ne^{1-x_n-\frac{ay_n}{x_n+y_n}}, \\ y_{n+1}=y_ne^{\epsilon \left( \frac{x_n}{x_n+y_n}-\delta \right) }, \end{array}\right. \end{aligned}$$

where the parameters $a>0,\delta >0, 0<\epsilon <1$ have the same meanings as in the system (4). We mainly consider in this paper the dynamical properties of the system (7).

The rest of this paper is organized as follows: In section “Existence and stability of fixed points”, we investigate the existence and stability of fixed points of the system (7). In section “Bifurcation analysis”, we derive the sufficient conditions for the transcritical bifurcation and the Neimark-Sacker bifurcation of the system (7) to occur. In section “Numerical simulation”, numerical simulations are performed to illustrate the theoretical results derived and reveal some new dynamical properties of the system.

Before we analyze the fixed points of the system (7), we recall the following lemma^3^.

### Lemma 1

Let $F(\lambda )=\lambda ^2+B\lambda +C$, where *B* and *C* are two real constants. Suppose $\lambda _1$ and $\lambda _2$ are two roots of $F(\lambda )=0$. Then the following statements hold.

(i) If $$F(1)>0,$$ then
  1. $|\lambda _1|<1$ and $|\lambda _2|<1$ if and only if $F(-1)>0$ and $C<1$;
  2. $\lambda _1=-1$ and $\lambda _2\ne -1$ if and only if $F(-1)=0$ and $B\ne 2$;
  3. $|\lambda _1|<1$ and $|\lambda _2|>1$ if and only if $F(-1)<0$;
  4. $|\lambda _1|>1$ and $|\lambda _2|>1$ if and only if $F(-1)>0$ and $C>1$;
  5. $\lambda _1$ and $\lambda _2$ are a pair of conjugate complex roots and, $|\lambda _1|=|\lambda _2|=1$ if and only if $-2<B<2$ and $C=1;$
  6. $\lambda _1=\lambda _2=-1$ if and only if $F(-1)=0$ and $B=2$.
(ii) If $F(1)=0,$ namely, 1 is one root of $F(\lambda )=0$, then the another root $\lambda$ satisfies $|\lambda |=(<,>)1$ if and only if $|C|=(<,>)1.$
(iii) If $F(1)<0,$ then $F(\lambda )=0$ has one root lying in $(1,\infty )$. Moreover,
  1. the other root $\lambda$ satisfies $\lambda <(=)-1$ if and only if $F(-1)<(=)0$;
  2. the other root $-1<\lambda <1$ if and only if $F(-1)>0$.

## Existence and stability of fixed points

In this section, we first consider the existence of fixed points and then analyze the local stability of each fixed point of the system (7).

The fixed points of the system (7) satisfy

$$\begin{aligned} x=xe^{1-x-\frac{ay}{x+y}}, y=ye^{\epsilon (\frac{x}{x+y}-\delta )}. \end{aligned}$$

Considering the biological meanings of the system (7), one only takes into account nonnegative fixed points. Thereout, one notices that the system (7) has and only has three nonnegative fixed points $E_0=(0, 0), E_1=(1, 0)$ and $E_2=(x_0,y_0)$ for $\max \{0,\frac{a-1}{a}\}<\delta <1$, where

$$\begin{aligned} x_0=1-a +a\delta ,\quad y_0=\frac{(1-a+a\delta )(1-\delta )}{\delta }. \end{aligned}$$

The Jacobian matrix of the system (7) at any fixed point *E*(*x*, *y*) takes the following form

$$\begin{aligned}J(E)=\begin{pmatrix} \Big [1-x+\frac{axy}{(x+y)^2}\Big ]e^{1-x-\frac{ay}{x+y}} &{} -\frac{ax^2}{(x+y)^2}e^{1-x-\frac{ay}{x+y}}\\ \frac{\epsilon y^2}{(x+y)^2}e^{\epsilon (\frac{x}{x+y}-\delta )} &{} \Big [1-\frac{\epsilon xy}{(x+y)^2}\Big ]e^{\epsilon (\frac{x}{x+y}-\delta )} \end{pmatrix}.\end{aligned}$$

The characteristic polynomial of Jacobian matrix *J*(*E*) reads

$$\begin{aligned} F(\lambda )=\lambda ^2-p\lambda +q, \end{aligned}$$

where

$$\begin{aligned} p&=Tr(J(E))=\Big (1-x+\frac{axy}{(x+y)^2}\Big )e^{1-x-\frac{ay}{x+y}}+\Big (1-\frac{\epsilon xy}{(x+y)^2}\Big )e^{\epsilon (\frac{x}{x+y}-\delta )},\\ q&=Det(J(E))=\Big [1-x+\frac{xy(\epsilon (x-1)+a)}{(x+y)^{2}}\Big ]e^{1-x-\epsilon \delta +\frac{\epsilon x-ay}{(x+y)}}. \end{aligned}$$

For the stability of fixed points $$E_0$$, $$E_1 $$ and $$ E_2$$, we can easily get the following Theorems 1, 2 and  3 respectively.

### Theorem 1

The fixed point $E_0=(0,0)$ of the system (7) is a saddle.

### Theorem 2

The following statements about the fixed point $E_1=(1,0)$ of the system (7) are true.

1. If $\delta <1$, then $E_1$ is a saddle.
2. If $\delta =1$, then $E_1$ is non-hyperbolic.
3. If $\delta >1$, then $E_1$ is a stable node.

The proofs for Theorems 1 and 2 are easy and omitted here.

### Theorem 3

When $\max \{0,\frac{a-1}{a}\}<\delta <1$, $E_2=(1-a+a\delta , \frac{(1-a+a\delta )(1-\delta )}{\delta })$ is a positive fixed point of the system (7). Let $\epsilon _{0}=\frac{a(1-\delta )(1+\delta )-1}{a\delta (1-\delta )^2}$ and ${\delta }_0$ be the unique positive root of the function $f(\delta ) = {\delta }^3- {\delta }^2+ \delta - \frac{a-1}{a}$ for $a>1$ and $\delta \in (\frac{a-1}{a}, \sqrt{\frac{a-1}{a}} )$. Then the following statements are true about the positive fixed point $E_2.$

1. When $0<a \le 1$, $E_2$ is a sink.
2. When $a \ge 1$, the folowing consequences hold.
  - If $\frac{a-1}{a} \le \delta \le {\delta }_0$, then $E_2$ is a source.
  (b) If ${\delta }_0 \le \delta \le \sqrt{\frac{a-1}{a}}$, then one further has:
    (i) for 0 < ε < ε_0_, *E*_2_ is a source;
    (ii) for ε = ε_0_, *E*_2_ is non-hyperbolic;
    (iii) for ε_0_< ε < 1, *E*_2_ is a sink.
  (c) If $\sqrt{\frac{a-1}{a}} \le \delta <1$, then $E_2$ is a sink.

### Proof

The Jacobian matrix of the system (7) at the fixed point $E_2$ can be simplified into

$$\begin{aligned} J({E_2})=\begin{pmatrix} a(1-{\delta }^2) &{} -a{\delta }^2\\ \epsilon (1-\delta )^2 &{} 1-\epsilon \delta (1-\delta ) \end{pmatrix}. \end{aligned}$$

The characteristic polynomial of Jacobian matrix $J({E_2})$ reads as

$$\begin{aligned} F(\lambda )=\lambda ^2-p\lambda +q, \end{aligned}$$

where

$$\begin{aligned} p= 1+(1-\delta )(a(1+\delta )-\epsilon \delta )\; \text {and} \; q=a(1-\delta )[1+\delta -\epsilon \delta (1-\delta )]. \end{aligned}$$

By calculating we get

$$\begin{aligned} F(1)=\epsilon \delta (1-\delta )[1-a(1-\delta )]>0 \end{aligned}$$

and

$$\begin{aligned} F(-1)&= 2+2a(1-{\delta }^2)-\epsilon \delta (1-\delta )[1+a(1-\delta )]\\&\quad> 2+2a(1-{\delta }^2)-\delta (1-\delta )[1+a(1-\delta )]\\&= 2-\delta + {\delta }^2 +a(1-\delta )(2+\delta +{\delta }^2)\\&\quad >0. \end{aligned}$$

Notice that

$$\begin{aligned} q> (=,< )1&\Leftrightarrow a(1-{\delta }^2)-a\epsilon \delta (1-\delta )^2>(=,<) 1 \\&\Leftrightarrow a\epsilon \delta (1-\delta )^2<(=,>) a(1-{\delta }^2)-1\\&\Leftrightarrow \epsilon<(=,>) \frac{a(1-{\delta }^2)-1}{a\delta (1-\delta )^2}\\&\Leftrightarrow \epsilon <(=, >) {\epsilon }_0, \end{aligned}$$

$$\begin{aligned} {\epsilon }_0 \le (>)0 \Leftrightarrow {\delta }^2 \ge (<) \frac{a-1}{a}, \end{aligned}$$

and

$$\begin{aligned} {\epsilon }_0 \ge (<)1 \Leftrightarrow f(\delta ) = {\delta }^3- {\delta }^2+ \delta - \frac{a-1}{a} \le (>)0. \end{aligned}$$

So, when $0<a \le 1$, $\frac{a-1}{a} \le 0 <{\delta }^2$. Equivalently, $\epsilon _{0}< 0<\epsilon$. Then $q<1$. By Lemma 1 (*i*.1) , $|\lambda _1|<1$ and $|\lambda _2|<1$, therefore, $E_2$ is a sink.

When $a > 1$, if $\frac{a-1}{a}< \delta \le {\delta }_0$, then $f(\delta ) \le f({\delta }_0)=0$, so, $\epsilon _{0}\ge 1 >\epsilon$, indicating $q>1$. In view of Lemma 1 (*i*.4) , $|\lambda _1|>1$ and $|\lambda _2|>1$, therefore $E_2$ is a source. If ${\delta }_0<\delta <\sqrt{\frac{a-1}{a}}$, then $0<\epsilon _{0}< 1$. Hence, for $0<\epsilon <\epsilon _{0}$, $q>1$. Lemma 1 (i.4) tells us that $E_2$ is a source. For $\epsilon =\epsilon _{0}$, $q=1$, $-2<p<2$. Lemma 1 (i.5) reads that Eq. (2.1) has a pair of conjugate complex roots $\lambda _1$ and $\lambda _2$ with $|\lambda _1|=|\lambda _2|=1$, implying $E_2$ is non-hyperbolic. For $\epsilon _{0}<\epsilon <1$, $q<1$. It follows from Lemma 1 (i.1) that $E_2$ is a sink.

If $\sqrt{\frac{a-1}{a}} \le \delta <1$, then $\epsilon _{0} \le 0 <\epsilon$. Hence, $q<1$. By Lemma 1 (i.1) one sees that $E_2$ is a sink.

The proof is finished. $\square$

## Bifurcation analysis

In this section, we use the center manifold theorem and bifurcation theory to analyze the local bifurcation problems of the system (7) at the fixed points $E_1$ and $E_2$, rspectively. For related work, refer to^6–12^.

### Bifurcation at $E_1$–Transcritical bifurcation

Theorem 2 shows that a bifurcation of the system (7) at the fixed point $$E_{1} $$ may occur in the space of parameters $$(a,\delta ,\epsilon ) \in S_{E_{1}}=\{(a,\delta ,\epsilon ) \in R^3_{+}| a>0, \delta >0, 0<\epsilon <1 \}$$. In fact, one has the following result.

#### Theorem 4

Suppose the parameters $(a,\delta ,\epsilon ) \in S_{E_{1}}$. Let $\delta _0=\frac{r}{a+1}$, then the system (7) undergoes a transcritical bifurcation at the fixed point $E_1$ when the parameter $\delta$ varies in a small neighborhood of the critical value $\delta _0$.

#### Proof

In order to show the detailed process, we proceed according to the following steps.

Let $u_n=x_n-1,v_n=y_n-0$, which transforms the fixed point $E_1=(1,0)$ to the origin *O*(0, 0), and the system (7) to

8 $$\begin{aligned} \left\{ \begin{array}{ll} u_{n+1}=(u_n+1)e^{-u_n-\frac{av_n}{u_n+1+v_n}}-1, \\ v_{n+1}=v_ne^{\epsilon (\frac{u_n+1}{u_n+1+y_n}-\delta )}. \end{array}\right. \end{aligned}$$

Giving a small perturbation ${\delta }^*$ of the parameter $\delta$, i.e., ${\delta }^*= \delta -{\delta }_0$, with $0<| {\delta }^* |\ll 1$, the system (8) is perturbed into

9 $$\begin{aligned} \left\{ \begin{array}{ll} u_{n+1}=(u_n+1)e^{-u_n-\frac{av_n}{u_n+1+v_n}}-1, \\ v_{n+1}=v_ne^{\epsilon (\frac{u_n+1}{u_n+1+y_n}-{\delta _{n}}^*-{\delta }_0)}. \end{array}\right. \end{aligned}$$

Letting $\delta _{n+1}^*=\delta _n^*=\delta ^*,$ the system (9) can be written as

10 $$\begin{aligned} \left\{ \begin{array}{ll} u_{n+1}=(u_n+1)e^{-u_n-\frac{av_n}{u_n+1+v_n}}-1, \\ v_{n+1}=v_ne^{\epsilon (\frac{u_n+1}{u_n+1+y_n}-{\delta _{n}}^*-{\delta }_0)},\\ \delta _{n+1}^*=\delta _n^*. \end{array}\right. \end{aligned}$$

Taylor expanding of the system (10) at $(u_n,v_n,{\delta }_n^*)=(0,0,0)$ takes the form

11 $$\begin{aligned} \left\{ \begin{array}{ll} u_{n+1}=&{}a_{100}u_n+a_{010}v_n+a_{200}u_n^{2}+a_{020}v_n^{2} +a_{110}u_nv_n\\ {} &{}+a_{300}u_n^{3}+a_{030}v_n^{3}+a_{210}u_n^{2}v_n+a_{120}u_nv_n^{2}+o(\rho _1^3), \\ v_{n+1}=&{}b_{100}u_n+b_{010}v_n+b_{001}b_n^*+b_{200}u_n^{2}+b_{020}v_n^{2}\\ &{}{+b_{002}{b_n^*}^{2}}+b_{110}u_nv_n+b_{101}u_nb_n^*+b_{011}v_nb_n^*\\ &{}+b_{300}u_n^{3}+b_{030}v_n^{3}+b_{003}{b_n^*}^{3}+b_{210}u_n^{2}v_n\\ &{}+b_{120}u_nv_n^{2}+b_{021}v_n^{2}b_n^*+b_{201}u_n^{2}b_n^*+b_{102}u_n{b_n^*}^{2}\\ &{}+b_{012}v_n{b_n^*}^{2}+b_{111}u_nv_nb_n^*+o(\rho _1^3),\\ {\delta }_{n+1}^*=&{}\delta _n^*, \end{array}\right. \end{aligned}$$

where $$\rho _1=\sqrt{u_n^2+v_n^2+(\delta _n^*)^2},$$

$$\begin{aligned} a_{100}&=0,a_{010}=-a,a_{200}=-1,a_{020}=a^2+2a,a_{110}=a,\\ a_{300}&=2, a_{030}=-a^3-6a^2-6a,a_{210}=-2a,a_{120}=-2a^2-4a,\\ b_{010}&=1,b_{020}=-2\epsilon ,b_{011}=-\epsilon ,b_{030}=3{\epsilon }^2+6\epsilon ,b_{120}=2\epsilon ,\\ b_{021}&=2{\epsilon }^2,b_{012}={\epsilon }^2,b_{100}=b_{001}=b_{200}=b_{002}=b_{110}=0,\\ b_{101}&=b_{300}=b_{003}=b_{210}=b_{201}=b_{102}=b_{111}=0.\\ \end{aligned}$$

Taking the transformation $(u_{n},v_{n},\delta _n^*)^{T}=T(X_{n},Y_{n},b_n)^{T}$ with $$ T=\begin{pmatrix} 1 &{} -a &{} 0\\ 0 &{} 1 &{} 0\\ 0 &{} 0 &{} 1 \end{pmatrix}$$, the system (11) is changed into the following form

12 $$\begin{aligned} \left\{ \begin{array}{ll} X_{n+1}=F(X_{n},Y_{n},b_n) +o(\rho _2^3),\\ Y_{n+1}=Y_{n}+G(X_{n},Y_{n},b_n)+o(\rho _2^3),\\ b_{n+1}=b_n, \end{array}\right. \end{aligned}$$

where $$ \rho _2 =\sqrt{X_{n}^{2}+Y_{n}^{2}+b_n^{2}}$$,

$$\begin{aligned} F(X_{n},Y_{n},b_n)&=m_{200}X_n^{2}+m_{020}Y_n^{2}+m_{002}{b_n}^{2}+m_{110}X_nY_n+m_{101}X_nb_n\\&\qquad +m_{011}Y_nb_n+m_{300}X_n^{3}+m_{030}Y_n^{3}+m_{003}{b_n}^{3}+m_{210}X_n^{2}Y_n\\&\qquad +m_{120}X_nY_n^{2}+m_{201}X_n^{2}b_n+m_{102}X_n{b_n}^{2}+m_{021}Y_n^{2}b_n\\&\qquad +m_{012}Y_n{b_n}^{2}+m_{111}X_nY_n b_n,\\ G(X_{n},Y_{n},b_n)&=l_{200}X_n^{2}+l_{020}Y_n^{2}+l_{002}{b_n}^{2}+l_{110}X_nY_n+l_{101}X_nb_n\\&\qquad +l_{011}Y_nb_n+l_{300}X_n^{3}+l_{030}Y_n^{3}+l_{003}{b_n}^{3}+l_{210}X_n^{2}Y_n\\&\qquad +l_{120}X_nY_n^{2}+l_{201}X_n^{2}b_n+l_{102}X_n{b_n}^{2}+l_{021}Y_n^{2}b_n\\&\qquad +l_{012}Y_n{b_n}^{2}+l_{111}X_nY_n b_n,\\ m_{200}=\,&-1,m_{020}=-a^2-2a\epsilon +2a,m_{002}=0,m_{110}=3a,m_{101}=0,\\ m_{011}=\,&-a\epsilon ,m_{300}=2,m_{030}=-3a^3-2a^2-6a+3a{\epsilon }^2+6a{\epsilon }-2a^2\epsilon ,\\m_{003}=&0,m_{210}=-8a, m_{120}=\,8a^2+2a\epsilon -4a,m_{201}=0,m_{102}=0,\\m_{021}=&2a{\epsilon }^2,m_{012}=a{\epsilon }^2, m_{111}=0,\\ l_{200}=\,&l_{002}=0,l_{020}=-2\epsilon ,l_{110}=0,l_{101}=0, l_{011}=-\epsilon ,l_{300}=0,\\ l_{030}=\,&3{\epsilon }^2+6\epsilon -2a\epsilon , l_{003}=0,l_{210}=0, l_{120}=2\epsilon ,l_{201}=0,l_{102}=0,\\ l_{021}=\,&2{\epsilon }^2,l_{012}={\epsilon }^2,l_{111}=0. \end{aligned}$$

Suppose on the center manifold

$$\begin{aligned} X_n=h(Y_n,b_n)=h_{20}Y_n^2+h_{11}Y_nb_n+h_{02}b_n^2+o(\rho _3^2), \end{aligned}$$

where $\rho _3=\sqrt{Y_n^2+b_n^2}$, then, according to

$$\begin{aligned} X_{n+1}=\,&F(h(Y_n,b_n),Y_{n},b_n)+o(\rho _3^2),\\ h(Y_{n+1},b_{n+1})=\,&h_{20}Y_{n+1}^2+h_{11}Y_{n+1}b_{n+1}+h_{02}b_{n+1}^2+o(\rho _3^2)\\ =\,&h_{20}{(Y_{n}+G(X_{n},Y_{n},b_n))}^{2}+h_{11}{(Y_{n}+G(X_{n},Y_{n},b_n))}b_n\\&\quad +h_{02}b_n^2+o(\rho _3^2), \end{aligned}$$

and $$ X_{n+1}=h(Y_{n+1},b_{n+1}),$$ we obtain the center manifold equation

$$\begin{aligned} F(h(Y_n,b_n),Y_{n},b_n)&\,=h_{20}{(Y_{n}+G(h(Y_n,b_n),Y_{n},b_n))}^{2}\\&\quad +h_{11}{(Y_{n}+G(h(Y_n,b_n),Y_{n},b_n))}b_n\\&\quad +h_{02}b_n^2+o(\rho _3^2). \end{aligned}$$

Comparing the corresponding coefficients of terms with the same orders in the above center manifold equation, we get

$$\begin{aligned} h_{20}=2a-a^2-2a\epsilon ,\quad h_{11}=-a\epsilon , \quad h_{02}=0. \end{aligned}$$

So, the system (12) restricted to the center manifold takes as

$$\begin{aligned} Y_{n+1}&=f(Y_n,b_n):=Y_{n}+G(h(Y_n,b_n),Y_{n},b_n)+o(\rho _3^2)\\&=Y_n-2\epsilon Y_{n}^2-\epsilon Y_{n} b_{n}+2{\epsilon }^2Y_{n}^2 b_{n}\\&\quad +{\epsilon }^2Y_{n} b_{n}^2+(3{\epsilon }^2+6\epsilon -2a\epsilon )Y_{n}^3+o(\rho _3^3). \end{aligned}$$

Therefore one has

$$\begin{aligned} f(Y_n,\delta _n)\bigg |_{(0,0)}&=0,\quad \frac{\partial f}{\partial Y_n}\bigg |_{(0,0)}=1,\quad \frac{\partial f}{\partial \delta _n}\bigg |_{(0,0)}=0,\\ \frac{\partial ^2f}{\partial Y_n\partial \delta _n}\bigg |_{(0,0)}&=-\epsilon \ne 0,\quad \frac{\partial ^2f}{\partial Y_n^2}\bigg |_{(0,0)}=-2\epsilon \ne 0. \end{aligned}$$

According to (21.1.42)–(21.1.46) in the literature^4^, or refer to^5^, all the conditions for the occurrence of a transcritical bifurcation are established, hence, there is an occurrence of a transcritical bifurcation for the system (7) at the fixed point $E_{1}$. The proof is over. $\square$

### Bifurcation at $E_2$–Neimark-Sacker bifurcation

First from the proof process of Theorem 3, we know that $F(1)>0$ and $F(-1)>0$. Namely, 1 and $-1$ are not eigenvalues of the system (7) at the fixed point $E_2$. So, it is impossible for the system (7) at the fixed point $E_2$ to undergo a transcritical bifurcation, or a fold bifurcation, or a pitchfork bifurcation or a flip bifurcation.

But, from Theorem 3 one also sees that, when $a >1$, ${\delta }_0<\delta <\sqrt{\frac{a-1}{a}}$, $\epsilon =\epsilon _{0}=\frac{a(1-\delta ^2)-1}{a\delta (1-\delta )^2}$, $E_2$ is non-hyperbolic. Moreover, when the parameter $\epsilon$ crosses the critical value $\epsilon _{0}$, the dimensional numbers of the stable manifold and the unstable manifold of the system (7) at the fixed point $E_{2}$ have an essential change. So, a bifurcation will occur at this time. Indeed, we derive that the system (7) at the fixed point $E_{2}$ will undergo a Neimark-Sacker bifurcation in the space of parameters

$$\begin{aligned} (a,\delta ,\epsilon ) \in S_{E_{+}} = \left\{ (a,\delta ,\epsilon ) \in R^3_{+}|a>1, {\delta }_0<\delta<\sqrt{\frac{a-1}{a}}, 0<\epsilon <1 \right\} , \end{aligned}$$

where ${\delta }_0$ is the unique positive root of the function $f(\delta ) = {\delta }^3- {\delta }^2+ \delta - \frac{a-1}{a}$ for $a>1$ and $\delta \in (\frac{a-1}{a}, \sqrt{\frac{a-1}{a}})$.

In order to show the process clearly, we carry out the following steps.

Take the change of variables $u_n=x_n-x_{0},v_n=y_n-y_{0}$, transforming the fixed point $E_2=(x_{0},y_{0})$ to the origin *O*(0, 0) and the system (7) into

13 $$\begin{aligned} \left\{ \begin{array}{ll} u_{n+1}=(u_n+x_{0})e^{1-u_n-x_{0}-\frac{a(v_n+y_0)}{u_n+x_0+v_n+y_0}}-x_{0}, \\ v_{n+1}=(u_n+y_{0})e^{\epsilon \left( \frac{x_0+u_n}{u_n+x_0+v_n+y_0}-\delta \right) }-y_{0}. \end{array}\right. \end{aligned}$$

Give a small perturbation $\epsilon ^*$ of the parameter $\epsilon$ around the critical value $\epsilon _{0}$, i.e., $\epsilon ^*= \epsilon -\epsilon _0$, then the perturbation of the system (13) can be regarded as follows

14 $$\begin{aligned} \left\{ \begin{array}{ll} u_{n+1}=(u_n+x_{0})e^{1-u_n-x_{0}-\frac{a(v_n+y_0)}{u_n+x_0+v_n+y_0}}-x_{0}, \\ v_{n+1}=(u_n+y_{0})e^{\left( \epsilon ^*+\epsilon _0 \right) \left( \frac{x_0+u_n}{u_n+x_0+v_n+y_0}-\delta \right) }-y_{0}. \end{array}\right. \end{aligned}$$

The corresponding characteristic equation of the linearized equation of the system (14) at the equilibrium point (0,0) can be expressed as

$$\begin{aligned} F(\lambda )=\lambda ^{2}-p(\epsilon ^*)\lambda +q(\epsilon ^*) =0, \end{aligned}$$

where

$$\begin{aligned} p(\epsilon ^*)=1+(1-\delta )(a(1+\delta )-\delta (\epsilon ^*+\epsilon _0)), \end{aligned}$$

and

$$\begin{aligned} q(\epsilon ^*)= a(1-\delta ) \left[ 1+\delta -\delta (1-\delta )(\epsilon ^*+\epsilon _0)\right] . \end{aligned}$$

Noticing $$ {p(\epsilon ^*)}\big |_{\epsilon ^*=0}=a(1-\delta ^2)+\frac{1}{a(1-\delta )}-\delta $$ and $$ {q(\epsilon ^*)}\big |_{\epsilon ^*=0}=1$$, one finds that $$p^2(0)-4q(0)<0 $$ always holds. In fact, it is easy to see

$$\begin{aligned} p^2(0)-4q(0)<0 \Leftrightarrow |p(0)|<2 \Leftrightarrow -2<a(1-\delta ^2)+\frac{1}{a(1-\delta )}-\delta <2. \end{aligned}$$

It follows from $a>0$ and $\delta \in (0, 1)$ that $-2<a(1-\delta ^2)+\frac{1}{a(1-\delta )}-\delta$. Whereas $a(1-\delta ^2)+\frac{1}{a(1-\delta )}-\delta<2 \Leftrightarrow a^2(1-\delta )^2(1+\delta ))-a(1-\delta )(2+\delta ) +1<0 \Leftrightarrow \frac{1}{(1-\delta )(1+\delta )}<a< \frac{1}{1-\delta } \Leftrightarrow \frac{a-1}{a}< \delta <\sqrt{\frac{a-1}{a}}$. This is verified by $(a,\delta ,\epsilon ) \in S_{E_{+}}$. So, when $0<|\delta ^*| \ll 1$, the two roots of $F(\lambda )=0$ take as

$$\begin{aligned} \lambda _{1,2}(\epsilon ^*)=\frac{p(\epsilon ^*)\pm \sqrt{p^2(\epsilon ^*)-4q(\epsilon ^*)}}{2}=\frac{p(\epsilon ^*)\pm i\sqrt{4q(\epsilon ^*)-p^2(\epsilon ^*)}}{2}, \end{aligned}$$

where *i* is an imaginary unit, namely, $i^2=-1$; moreover

$$\begin{aligned} (|\lambda _{1,2}(\epsilon ^*)|)\big |_{\epsilon ^*=0}=\sqrt{q(\epsilon ^*)}\big |_{\epsilon ^*=0}=\sqrt{a(1-\delta )(1+\delta -\epsilon _0\delta (1-\delta ))}=1, \end{aligned}$$

which implies

$$\begin{aligned} \Big (\frac{d|\lambda _{1,2}(\epsilon ^*)|}{d\epsilon ^*}\Big )\bigg |_{\epsilon ^*=0}= -\frac{1}{2}a\delta (1-\delta )^2<0. \end{aligned}$$

The occurrence of Neimark-Sacker bifurcation requires the following two conditions to be satisfied

$$\begin{aligned}{} & {} (H.1) \quad \Big (\frac{d|\lambda _{1,2}(\epsilon ^*)|}{d\epsilon ^*}\Big )\bigg |_{\epsilon ^*=0}\ne 0;\\{} & {} (H.2) \quad \lambda _{1, 2}^{i}(0)\ne 1,i=1,2,3,4. \end{aligned}$$

The transversal condition $H_1$ obviously holds. For the sake of convenience in discussing the nondegenerate condition ($H_2$), let

$$\begin{aligned} \begin{array}{ll} \alpha _1=\frac{a^2(1-\delta ^2)(1-\delta )-a\delta (1-\delta )+1}{2a(1-\delta )},\\ \alpha _2=\frac{\sqrt{4a^2(1-\delta )^2-(a^2(1-\delta ^2)(1-\delta )-a\delta (1-\delta )+1)^2}}{2a(1-\delta )}. \end{array} \end{aligned}$$

Then $\lambda _{1,2}(0)=\alpha _1\pm \alpha _2i.$ It is easy to derive $\lambda _{1, 2}^m(0)\ne 1$ for all $m=1,2,3,4$. Hence, (H.1) and (H.2) hold. According to [4, page 517–522], all the conditions for a Neimark-Sacker bifurcation to occur are satisfied.

In order to derive the normal form of the system (14), we expand the system (14) in power series to the third-order term at the origin to obtain

15 $$\begin{aligned} \left\{ \begin{array}{ll} u_{n+1}=&{}c_{10}u_n+c_{01}v_n+c_{20}u_n^{2}+c_{11}u_nv_n+c_{02}v_n^{2}\\ {} &{}+c_{30}u_n^{3}+c_{21}u_n^{2}v_n+c_{12}u_nv_n^{2}+c_{03}v_n^{3}+o(\rho _4^3),\\ v_{n+1}=&{}d_{10}u_n+d_{01}v_n+d_{20}u_n^{2}+d_{11}u_nv_n+d_{02}v_n^{2}\\ {} &{}+d_{30}u_n^{3}+d_{21}u_n^{2}v_n+d_{12}u_nv_n^{2}+d_{03}v_n^{3}+o(\rho _4^3) \end{array}\right. \end{aligned}$$

where $$\rho _4=\sqrt{u_n^2+v_n^2},$$

$$\begin{aligned} c_{10}&=a(1-\delta ^2) ,c_{01}=-a{\delta }^2,c_{11}=-\frac{a\delta ^2(a(1-\delta ^2)+1-2\delta )}{1-a(1-\delta )},\\ c_{20}&=-1-\frac{a\delta (1-\delta )(2\delta -1-a(1-\delta ^2))}{1-a(1-\delta )},\\ c_{02}&=\frac{a\delta ^3(2+a\delta )}{1-a(1-\delta )},c_{03}=\frac{a\delta ^4}{(1-a(1-\delta ))^2}\big [a^2\delta ^2+6a\delta +6\big ],\\ c_{30}&=1+\frac{a\delta (1-\delta )^2}{(1-a(1-\delta ))^2}\big [a^2(1+\delta )^2(1-\delta )+3a(1-3\delta )-2(1+3\delta )\big ],\\ c_{21}&=\frac{a\delta ^2}{(1-a(1-\delta ))^2}\big [-2\delta +a(1-\delta )+a^2\delta (1-\delta )^2(1+\delta )\\&\quad -2(1-\delta )(1-2\delta )(2+\delta )\big ]+a(6\delta -6\delta ^2-1),\\ c_{12}&=\frac{a\delta ^3}{(1-a(1-\delta ))^2}\big [2-6\delta +a(a\delta (1-\delta ^2)+6\delta -6\delta ^2-2)],\\ d_{10}&=\epsilon (1-\delta )^2,d_{01}=1-\delta \epsilon (1-\delta ),\\ d_{20}&=\frac{\epsilon \delta (1-\delta )^2}{1-a(1-\delta )}\big (\epsilon (1-\delta )-2\big ),d_{11}=\frac{\epsilon \delta ^2(1-\delta )}{1-a(1-\delta )}\big (2-\epsilon (1-\delta )\big ),\\ d_{02}&=\frac{\epsilon \delta ^3}{1-a(1-\delta )}\big (\epsilon (1-\delta )-2\big ),d_{30}=\frac{\epsilon (1-\delta )^2}{1-a(1-\delta )}\big ((\epsilon (1-\delta )-3)^2-3\big ),\\ d_{21}&=\frac{\epsilon \delta ^2(1-\delta )}{(1-a(1-\delta ))^2}\big (2(1-3\delta )+\epsilon \delta (1-\delta )(6\delta -1-\epsilon (1-\delta ))\big ),\\ d_{12}&=\frac{\epsilon \delta ^3}{(1-a(1-\delta ))^2}\big (6\delta -4-2\epsilon (2\delta -1)(1-\delta ^2)-\delta \epsilon ^2(1-\delta )^2\big ),\\ d_{03}&=\frac{\epsilon \delta ^4}{(1-a(1-\delta ))^2}\big (6+3\epsilon (2\delta -1)-\epsilon ^2\delta (1-2\delta )\big ).\\ \end{aligned}$$

Make a change of variables $$(u,v)^{T}=T(X,Y)^{T} $$ with matrix $$ T=\begin{pmatrix} a_{01}&{}0 \\ \alpha _1-a_{10}&{}-\alpha _2 \end{pmatrix},$$ then the system (15) is transformed as

16 $$\begin{aligned} \left\{ \begin{array}{ll} X\rightarrow &{}\alpha _1X -\alpha _2 Y+ {\overline{F}}(X,Y)+o(\rho _5^3),\\ Y\rightarrow &{}\alpha _2 X+ \alpha _1Y+ {\overline{G}}(X,Y)+o(\rho _5^3),\\ \end{array}\right. \end{aligned}$$

where $$\rho _5=\sqrt{X^2+Y^2},$$

$$\begin{aligned} {\overline{F}}(X,Y)&=e_{20}X^{2}+e_{11}XY+e_{02}Y^{2}+e_{30}X^{3}+e_{21}X^{2}Y+e_{12}XY^{2}+e_{03}Y^{3},\\ {\overline{G}}(X,Y)&=f_{20}X^{2}+f_{11}XY+f_{02}Y^{2}+f_{30}X^{3}+f_{21}X^{2}Y+f_{12}XY^{2}+f_{03}Y^{3},\\ e_{20}&=a_{01}a_{20}+a_{11}(\alpha _1-a_{10})+\frac{a_{02}}{a_{10}}(\alpha _1-a_{10})^2 ,e_{02}=\frac{\alpha _2^2a_{02}}{a_{01}},\\ e_{11}&=-\alpha _{2}a_{11}-\frac{2\alpha _2a_{02}}{a_{01}}(\alpha _1-a_{10}), e_{03}=-\frac{\alpha _2^3a_{03}}{a_{01}},\\ e_{30}&=a_{01}^2a_{30}+a_{01}a_{21}(\alpha _1-a_{10})+a_{12}(\alpha _1-a_{10})^2+\frac{a_{03}}{a_{01}}(\alpha _1-a_{10})^3,\\ e_{12}&=\alpha _2^2a_{12}+\frac{3a^2a_{03}}{a_{01}}(\alpha _1-a_{10}),\\ e_{21}&=-\alpha _2(a_{01}a_{21}+2a_{12}(\alpha _1-a_{10})+\frac{3a_{03}}{a_{01}}(\alpha _1-a_{10})^2),\\ f_{20}&=a_{01}^2\big (\frac{a_{20}(\alpha _1-a_{10})}{\alpha _2a_{10}}-\frac{b_{20}}{\alpha _1}\big )+a_{01}(\alpha _1-a_{10})\big (\frac{a_{11}(\alpha _1-a_{10})}{\alpha _2a_{10}}-\frac{b_{11}}{\alpha _1}\big )\\&\quad +(\alpha _1-a_{10})^2\big (\frac{a_{02}(\alpha _1-a_{10})}{\alpha _2a_{10}}-\frac{b_{02}}{\alpha _1}\big ),\\ f_{02}&=\alpha _2\big (\frac{\alpha _1-a_{10}}{a_{10}}-b_{02}\big ) ,f_{03}=\alpha _2^2b_{03}+\alpha _2^2a_{03}\frac{a_{10}-\alpha _1}{a_{10}},\\ f_{30}&=\frac{\alpha _1-a_{10}}{\alpha _2a_{10}}\big [a_{01}^3a_{30}+a_{01}^2a_{21}(\alpha _1-a_{10})+a_{01}a_{12}(\alpha _1-a_{10})^2+a_{03}(\alpha _1-a_{10})^3\big ]\\&\quad -\frac{1}{\alpha _1}\big [a_{01}^3b_{30}+a_{01}^2b_{21}(\alpha _1-a_{10})+a_{01}b_{12}(\alpha _1-a_{10})^2+b_{03}(\alpha _1-a_{10})^3\big ],\\ f_{11}&=a_{01}b_{11}+2b_{02}(\alpha _1-a_{10})+\frac{a_{10}-\alpha _1}{a_{10}}(a_{01}a_{11}+2a_{02}(\alpha _1-a_{10})),\\ f_{12}&=a_{01}\alpha _2\big (\frac{a_{12}(\alpha _1-a_{10})}{a_{10}}-b_{12}\big )+3\alpha _2(\alpha _1-a_{10})\bigg (\frac{a_{03}(\alpha _1-a_{10})}{a_{10}}-b_{03}\bigg ),\\ f_{21}&=3(\alpha _1-a_{10})^2\bigg (b_{02}+\frac{a_{03}(a_{10}-\alpha _1)}{a_{10}}\bigg )+2a_{01}(\alpha _1-a_{10})\bigg (b_{12}+\frac{a_{12}(a_{10}-\alpha _1)}{a_{10}}\bigg )\\&\quad +a_{01}^2\bigg (b_{21}+\frac{a_{21}(a_{10}-\alpha _1)}{a_{10}}\bigg ). \end{aligned}$$

Furthermore

$$\begin{aligned} {\overline{F}}_{XX}&=2e_{20}, {\overline{F}}_{XY}=e_{11}, {\overline{F}}_{XXX}=6e_{30}, {\overline{F}}_{YY}=2e_{02}, {\overline{F}}_{XXY}=2e_{21},\\ {\overline{F}}_{XYY}&=2e_{12}, {\overline{F}}_{YYY}=6e_{03}, {\overline{G}}_{XX}=2f_{20}, {\overline{G}}_{XY}=f_{11}, {\overline{G}}_{XXX}=6f_{30},\\ {\overline{G}}_{YY}&=2f_{02}, {\overline{G}}_{XXY}=2f_{21}, {\overline{G}}_{XYY}=2f_{12}, {\overline{G}}_{YYY}=6f_{03}. \end{aligned}$$

In order to determine the stability and direction of the Neimark-Sacker bifurcation, we need to calculate the discriminating quantity^4,5^

17 $$\begin{aligned} L=-Re\Bigl (\frac{(1-2\lambda _1)\lambda _2^2}{1-\lambda _1}\zeta _{20}\zeta _{11}\Bigr )-\frac{1}{2}|\zeta _{11}|^2-|\zeta _{02}|^2+ Re(\lambda _2\zeta _{21}), \end{aligned}$$

which is required not to equal zero, where

$$\begin{aligned} \zeta _{20}&=\frac{1}{8} \left[ {\overline{F}}_{XX}-{\overline{F}}_{YY}+2{\overline{G}}_{XY}+i \left( {\overline{G}}_{XX}-{\overline{G}}_{YY}- 2{\overline{F}}_{XY}\right) \right] ,\\ \zeta _{11}&=\frac{1}{4} \left[ {\overline{F}}_{XX}+{\overline{F}}_{YY}+i \left( {\overline{G}}_{XX}+{\overline{G}}_{YY} \right) \right] ,\\ \zeta _{02}&=\frac{1}{8} \left[ {\overline{F}}_{XX}-{\overline{F}}_{YY}-2{\overline{G}}_{XY} +i \left( {\overline{G}}_{XX}-{\overline{G}}_{YY}+2{\overline{F}}_{XY}\right) \right] ,\\ \zeta _{21}&=\frac{1}{16} \bigg [{\overline{F}}_{XXX}+{\overline{F}}_{XYY}+{\overline{G}}_{XXY}+{\overline{G}}_{YYY}+i \bigg ({\overline{G}}_{XXX}+{\overline{G}}_{XYY}-{\overline{F}}_{XXY}\\&\quad -{\overline{F}}_{YYY}\bigg )\bigg ]. \end{aligned}$$

Based on above analysis, we obtain the following conclusion.

#### Theorem 5

Assume the parameters *a*, $\delta$, $\epsilon$ in the space

$$\begin{aligned}(a,\delta ,\epsilon ) \in S_{E_{+}} = \left\{ (a,\delta ,\epsilon ) \in R^3_{+}|a>1, {\delta }_0<\delta<\sqrt{\frac{a-1}{a}}, 0<\epsilon <1 \right\} , \end{aligned}$$

where ${\delta }_0$ is the unique positive root of the function $f(\delta ) = {\delta }^3- {\delta }^2+ \delta - \frac{a-1}{a}$ for $a>1$ and $\delta \in \left( \frac{a-1}{a}, \sqrt{\frac{a-1}{a}} \right)$. Let $\epsilon _{0}=\frac{a(1-\delta ^2)-1}{a\delta (1-\delta )^2}$ and *L* be defined as above (17) . If the parameter $\epsilon$ varies in a small neighborhood of the critical value $\epsilon _0$, then the system (7) at the fixed point $E_2$ undergoes a Neimark-Sacker bifurcation. In addition, if $L<(or>)0$, then an attracting (or repelling) invariant closed curve bifurcates from the fixed point $E_2$ for $\epsilon >(or <) \epsilon _{0}$.

## Numerical simulation

In this section, with the help of Matlab software, we draw bifurcation diagrams, maximum Lyapunov exponents and phase portraits of the system (7) at the fixed point $E_2$ when the parameter $\epsilon$ varies. These sufficiently illustrate the above theoretical results derived, and further reveal some new dynamical behaviors to occur.

Vary $\epsilon$ in the range (0.19,0.6) and fix $a=1.4$, $\delta =0.5$ with the initial value $(x_0, y_0)=(0.6,0.2)$. It is easy to get the unique positive fixed point $E_2=(0.2728,0.2728)$ and $\epsilon _0 =0.5$, and the eigenvalues of $J(E_2)$ are $\lambda _{1,2} =0.81252149\pm 0.5829312498i$ with $|\lambda _{1,2}| = 1$.

**Figure 1:**
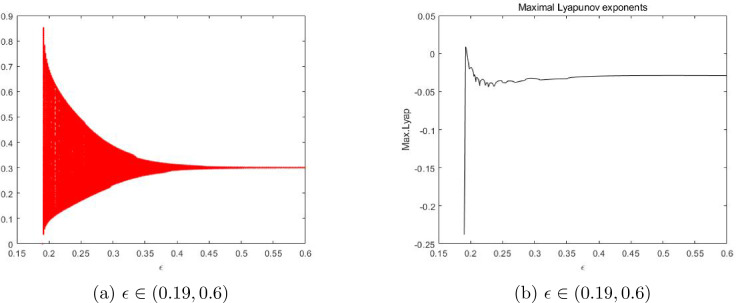
Bifurcation of the system (7) in $(\epsilon , x)$-plane and maximal Lyapunov exponents.

**Figure 2:**
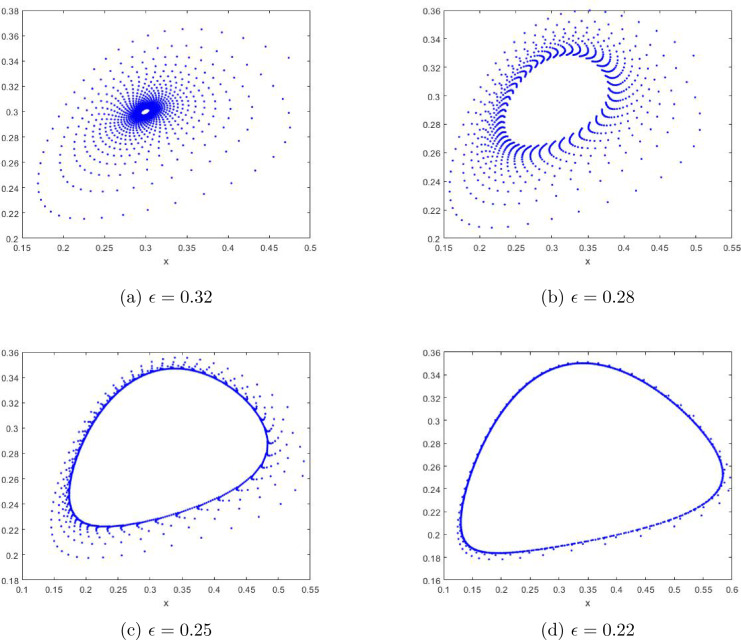
Phase portraits for the system (7) with $a=1.4$, $\delta =0.5$ and different $\epsilon$ with the initial value $(x_0,y_0)=(0.6,0.2)$ outside the closed orbit.

The bifurcation diagram in the $(\epsilon , x)$ plane is given in Fig. 1. It is easy to see that the fixed point $E_2$ is stable for $\epsilon >0.5$, and that a Neimark-Sacker bifurcation occurs when $\epsilon = 0.5$, and that the fixed point $E_2$ becomes unstable when $\epsilon <0.5$. Figure 1b depicts the corresponding maximum Lyapunov exponents, which are positive for the parameter $\epsilon \in (0.192, 0.198)$, which means the chaos occurs in the system (7) at this time. This is a new dynamical phenonmenon, which has not been theoretically verified yet.

**Figure 3:**
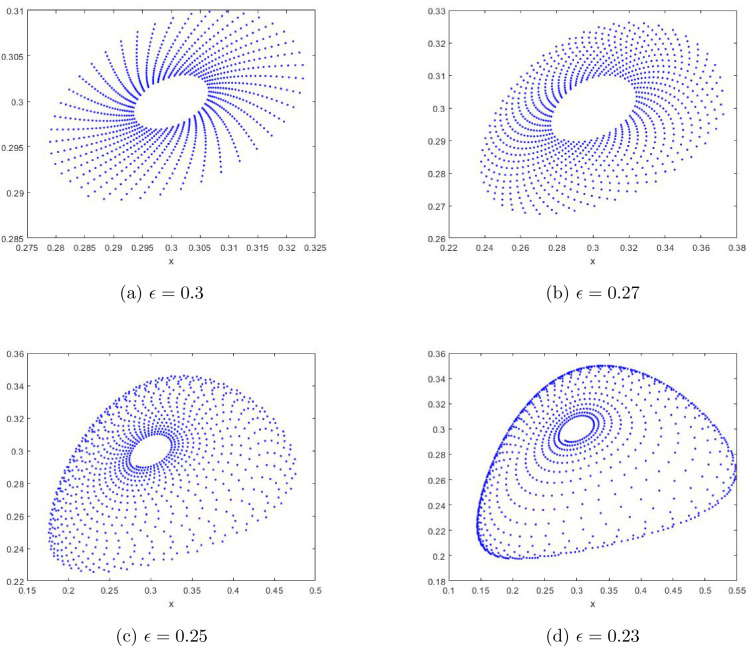
Phase portraits for the system (7) with $a=1.4$, $\delta =0.5$ and different $\epsilon$ with the initial value $(x_0,y_0)=(0.28,0.3)$ inside the closed orbit.

From the phase portraits in Figs. 2 and 3, we infer the stability of $E_{2}$, because Fig. 2a–d show that the closed curve is stable outside while Fig. 3a–d indicate that the closed curve is stable inside for the fixed point $E_{2}$ as long as the assumptions of Theorem 5 hold. This fits the conclusion of Theorem 5.

## Discussion and conclusion

In this paper, toward a derived discrete-time slow-fast predator-prey system with ratio-dependent functional response, under given parametric conditions, we completely state the existence and stability of three nonnegative equilibria. Especially for the positive equilibrium $E_2$, a complete classifaction is given in the whole parametric space of it existing.

What’s more important, we derive the sufficient conditions for transcritical bifurcation and Neimark-Sacker bifurcation of the system at the equilibria $$E_1$$ and $$E_2$$ to occur. Our results clearly display that, for $$a >1$$ and $$ {\delta }_0<\delta <\sqrt{\frac{a-1}{a}}$$, the positive equilibrium $$E_{2} $$ is asymptotically stable when $$ 0<\epsilon <\epsilon _{0}=\frac{a(1-\delta ^2)-1}{a\delta (1-\delta )^2} $$ and unstable when $$ \epsilon _0<\epsilon <1$$. Hence, the system (7) at the positive equilibrium $$E_{2}$$ undergoes a bifurcation, which has been shown to be a Neimark-Sacker bifurcation, when the parameter $$ \epsilon $$ goes through the critical value $$ \epsilon _{0} $$.

Numerical simulations not only confirm the theoretical analysis results, but also find some new properties of the system (7)—chaos occuring.

Our results also clearly demonstrate that the system (7) is very sensitive to its fast time scale parameter variable $\epsilon$, namely, to appropriately adjust the value of fast time scale parameter variable $\epsilon$ may alter the stability of the system (7). So, the results in this paper also provide a way for how to control the stability of the system (7).

## Data Availability

All data generated or analysed during this study are included in this published article.
